# Chemical Composition of Extracts from Various Parts of Feverfew (*Tanacetum parthenium* L.) and Their Antioxidant, Protective, and Antimicrobial Activities

**DOI:** 10.3390/ijms252212179

**Published:** 2024-11-13

**Authors:** Monika Michalak, Małgorzata Stryjecka, Paulina Żarnowiec, Martyna Zagórska-Dziok, Anna Kiełtyka-Dadasiewicz

**Affiliations:** 1 Department of Pharmaceutical Sciences, Collegium Medicum, Jan Kochanowski University, IX Wieków Kielc 19, 35-317 Kielce, Poland; 2 Department of Dietetics, The University College of Applied Sciences in Chełm, Pocztowa 54, 22-100 Chełm, Poland; mstryjecka@panschelm.edu.pl; 3 Garden of Cosmetics Plants and Raw Materials, Research and Science Innovation Centre, Tarasowa 4/96, 20-819 Lublin, Poland; anna.kieltyka-dadasiewicz@up.lublin.pl; 4 Department of Microbiology, Faculty of Natural Sciences, Jan Kochanowski University, Uniwersytecka 7, 25-406 Kielce, Poland; paulina.zarnowiec@ujk.edu.pl; 5 Department of Technology of Cosmetic and Pharmaceutical Products, Medical College, University of Information Technology and Management in Rzeszów, Sucharskiego 2, 35-225 Rzeszów, Poland; mazagorska@wsiz.edu.pl; 6 Department of Plant Production Technology and Commodity Sciences, University of Life Sciences in Lublin, Akademicka 15, 20-950 Lublin, Poland

**Keywords:** *Tanacetum parthenium*, plant extracts, essential oil, bioactive compounds, antioxidant properties, antimicrobial activity, protective effect

## Abstract

*Tanacetum parthenium* is a medicinal plant from the Asteraceae family that can be applied externally in the case of various skin diseases. The aim of the study was to perform a phytochemical analysis of hydroethanolic extracts from the aerial parts (herb), flower heads, and leaves of feverfew and to assess their biological properties. Hydrodistilled oils were analyzed using GC-MS. The chemical composition of the extracts was estimated using spectrophotometry and the HPLC method. Moreover, the extracts were evaluated to determine their antioxidant potential using DPPH and FRAP and measuring the intracellular level of ROS. The cytotoxicity of extracts toward keratinocytes and fibroblasts was also analyzed, as well as their antimicrobial properties against 12 microorganisms. The results of the research revealed that chrysanthenone and α-thujone were the dominant volatile compounds in the essential oil from the flowers, while camphor, trans-chrysanthenyl acetate, and camphene were predominant in the essential oil from the leaves and herb. The results of HPLC showed that the major polyphenol compounds present in the hydroethanolic extracts from various parts of *T. parthenium* were 3,5-dicaffeoyl-quinic acid, chlorogenic acid, and 3,4-dicaffeoyl-quinic acid. The extract from feverfew flowers was shown to have the highest content of total polyphenols, flavonoids, and phenolic acids, as well as the highest antioxidant potential. In turn, the herb extract had the highest content of condensed tannins and terpenoids and exhibited the most effective antimicrobial properties against the 12 bacterial and fungal strains. Moreover, the hydroethanolic extracts from different parts of *T. parthenium* plants were shown to have a potent protective effect on skin cells. The present study supports the potential applications of *Tanacetum parthenium* in the cosmetic and pharmaceutical industries.

## 1. Introduction

*Tanacetum parthenium* L., also known as *Chrysanthemum parthenium*, *Matricaria parthenium*, and *Leucanthemum parthenium* and by its common names featherfew, feverfew, bachelor’s button, and midsummer daisy, is a member of the Asteraceae family found in Australia, Europe, China, Japan, and North Africa. It is a perennial plant that reaches a height of 30–80 cm, with short, fine hairs on the stem and ovate, greenish-yellow, pinnate leaves. The flower heads at the tops of the shoots, with white ligulate flowers and yellow tubular flowers, measure up to 2 cm in diameter. All parts of the plant have a characteristic strong and pleasant spicy scent.

The pharmacological material is the herb, collected during the flowering stage, from June to September. *T. parthenium* has a long history of use in traditional medicine, in which two main types of application can be distinguished: analgesic (e.g., for fever, headache, rheumatic pain, and stomach ache) and gynecological (e.g., to reduce the risk of miscarriage or to regulate the menstrual cycle). In addition, it has been used to treat asthma, cough, insect bites, and worms, as well as skin diseases such as dermatitis and psoriasis [1,2]. Research to date indicates that the pharmacological properties of feverfew are linked to the chemical composition of the individual parts of this plant [1]. *T. parthenium* is chemically very complex, containing sesquiterpene lactones, monoterpenes, flavonoid glycosides, and other compounds. Among sesquiterpene lactones (which include eudesmanolides, germacranolides, and guaianolides), the principal compound is parthenolide, which comprises up to 85% of the total sesquiterpene content [1,3]. Other sesquiterpene lactones in *T. parthenium* include costunolide, 3-β-hydroxy parthenolide, secotanaparthenolide, artecanin, canin, and epoxysantamarin [1,2]. Flavonoids identified include tanetin, quercetin, apigenin, luteolin, chrysoeriol, santin, and centaureidin [4,5]. Volatile substances contained in the essential oil of feverfew include monoterpenes, such as camphor, camphene, ester derivatives of chrysanthenol, verbenol, and borneol, as well as sesquiterpene hydrocarbons such as germacrene D and β-farnesene [6]. It is now widely recognized that the fraction of sesquiterpene lactones, present mainly in the leaves of this plant, is the most important for biological activity and potential therapeutic uses [2]. For example, parthenolide, which is present in the greatest amount in the aerial parts (leaves and stems) of the plant during flowering, is considered to be a compound with a wide range of biological activities (e.g., anti-inflammatory, anti-migraine, and antibacterial) due to the presence of a conjugated double bond system in its structure [1,2]. Another example, contained mainly in the leaves, flowers, and seeds of feverfew, is the lipophilic flavonoid tanetin, which shows anti-inflammatory effects due to inhibition of prostaglandin production [4].

In the scientific literature, much attention is paid to research on the main component of *T. parthenium*, i.e., the sesquiterpene lactone parthenolide, and its properties and potential therapeutic applications, i.e., as a migraine prophylactic agent [7,8,9]. At the same time, up-to-date information on extracts from this plant is limited. There is a lack of data assessing the content of bioactive compounds in different parts of *T. parthenium*. The phytochemical composition of a plant extract and thus its biological properties may differ depending on the part of the plant from which it is prepared. Therefore, the aim of the present study was to assess the biologically active chemical compounds (total polyphenols, total flavonoids, total phenolic acids, condensed tannins, and terpenoids via spectrophotometric methods, as well as a qualitative and quantitative analysis of flavonoids and phenolic acids using HPLC) in hydroethanolic extracts from different parts of feverfew and their potential antioxidant, protective, and antimicrobial properties.

## 2. Results

### 2.1. Plant Material Characteristics

Feverfew forms a leafy stem with many branches and numerous small flower heads on the end of each branch. The feverfew herb, i.e., the top of the stem with the leaves and flowers, has long been used. The herb obtained for this study was divided into three parts: the leaves, the inflorescences with the pedicels, and the remainder. The flower heads made up, on average, 36.5% of the herb, and the leaves comprise 31.7% (Table 1). The remaining 31.8% was waste; these were mainly stems considered to be without value and not used for further analysis. The leaves had the highest fresh-to-dry ratio; their weight decreased more than 4.5 times after drying. The flower heads and herbs had similar ratios from 3.56 to 3.87.

### 2.2. Chemical Composition of Essential Oil (EO) Obtained from Different Parts of T. parthenium

**Figure 1:**
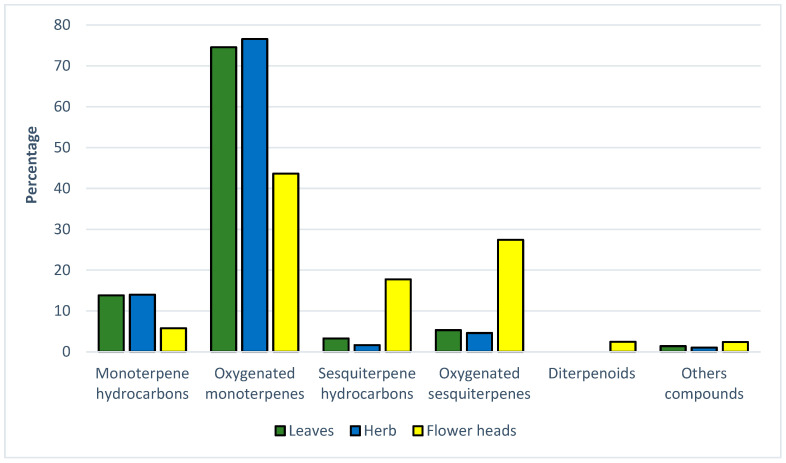
Comparison of the percentages of chemical groups identified in essential oils from *T. parthenium* leaves, herb, and flower heads.

The chemical composition of the oils isolated from different plant parts, i.e., the leaves, herb, and flower heads, was determined using GC/MS. An analysis of the essential oil of *T*. *parthenium* flower heads revealed the presence of 83 compounds, representing 99.37% of the total oil. The predominant components in the oil were oxygenated monoterpenes (43.61%), sesquiterpene hydrocarbons (17.75%), and oxygenated sesquiterpenes (27.40%). For the leaf oil, 61 compounds accounted for 98.37% of the total composition, while a total of 68 constituents were identified in the herb oil, accounting for 97.53% of the total composition. The EO from the leaves and herb contained large amounts of oxygenated monoterpenes (74.55% and 76.57%, respectively) and monoterpene hydrocarbons (13.83% and 13.99%, respectively) (Figure 1).

The content of the major individual constituents of the EOs obtained from the leaves and herb was similar, while different compounds were predominant in the oil from the flower heads. The most important of these are presented in Table 2. Details regarding the percentage shares of the components of the essential oils from different parts of feverfew are presented in the Supplementary Material (Table S1). The predominant compounds in the EO of *T*. *parthenium* flower heads were chrysanthenone (14.08%), α-thujone (9.60%), tetradecane (6.96%), camphor (6.30%), and α-cadinol (5.84%), while the dominant compounds in the EO from the leaves and herb were camphor (40.14% and 38.96%, respectively), trans-chrysanthenyl acetate (22.17% and 25.26%), and camphene (7.12% and 6.57%).

### 2.3. Phytochemical Constituents of Hydroethanolic Extracts Obtained from Different Parts of T. parthenium

In this study, spectrophotometric methods were used to assess the content of polyphenols, flavonoids, phenolic acids, condensed tannins, and terpenoids in hydroethanolic extracts from the leaves (TLE), herb (THE), and flower heads (TFE) of feverfew (Table 3). The results revealed that the extract obtained from the feverfew flowers had the highest content of total polyphenols (67.41 mg/mL), flavonoids (19.33 mg/mL), and phenolic acids (5.10 mg/mL), while the extract from the herb had the highest content of condensed tannins (22.89 mg/mL) and terpenoids (54.41 mg/mL). All differences were statistically significant at *p* < 0.05.

Qualitative analysis using HPLC-DAD showed the presence of four flavonoids and seven phenolic acids (Table 4). The content of these compounds differed statistically significantly depending on the plant part. Kaempferol-3-rutinoside was the predominant flavonoid in the extract from the herb (1.15 mg/mL), flower heads (0.57 mg/mL), and leaves (0.40 mg/mL). Santin, a flavonoid characteristic of feverfew, was detected and quantified in all of the extracts: 0.46, 0.42, and 0.32 mg/mL for the herb, flower heads, and leaves, respectively. The predominant phenolic acids identified in the extracts of all parts of the plant were 3,5-dicaffeoyl-quinic acid and chlorogenic acid. In addition, 3,4-dicaffeoyl-quinic acid was a predominant compound in the herb (2.65 mg/mL).

### 2.4. Cell Viability Assay

Cytotoxicity analyses of extracts from different parts of feverfew were performed using two tests. The first (Alamar Blue assay, AB) was based on the detection of the metabolic activity of cells (fibroblasts and keratinocytes) via fluorometric measurement of the reduction in the dye resazurin. The second test (neutral red assay, NR) measured the ability of fibroblasts and keratinocytes exposed to the extracts to take up the neutral red dye and incorporate it in the lysosomes. The uptake of neutral red is a measure of cell viability, and the degree of incorporation of the dye is measured spectrophotometrically.

**Figure 2:**
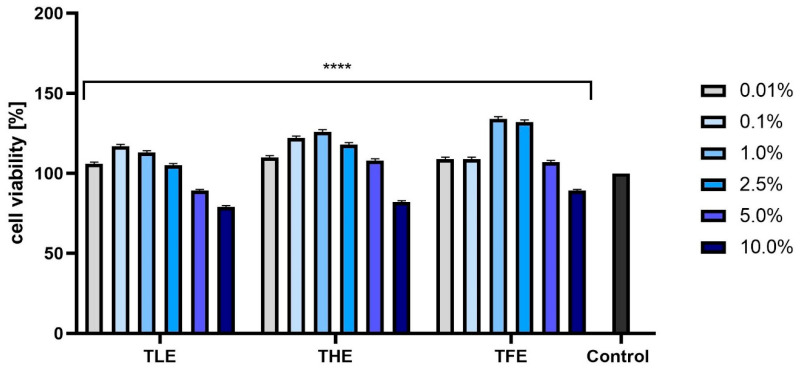
Effect of extracts from *Tanacetum parthenium* leaves (TLE), herb (THE), and flower heads (TFE) on resazurin reduction in fibroblasts (BJ) in the concentration range of 0.01–10.0%. The control consisted of BJ cells not exposed to any extract, for which viability was assumed to be 100%. Three independent experiments were performed, in which each concentration was tested in four replicates. **** *p* < 0.0001 versus the control.

**Figure 3:**
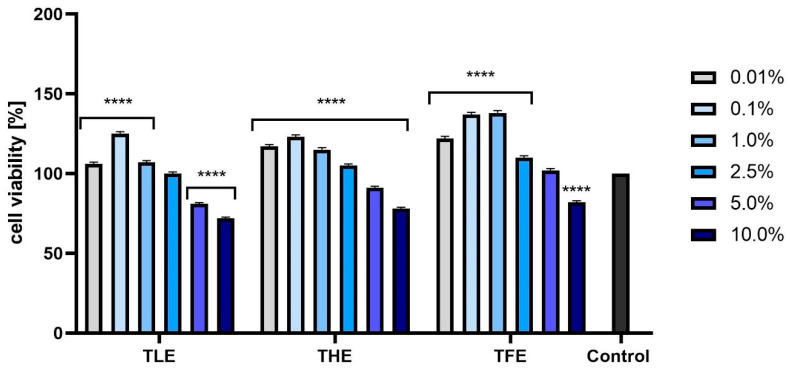
Effect of extracts from *Tanacetum parthenium* leaves (TLE), herb (THE), and flower heads (TFE) on resazurin reduction in keratinocytes (HaCaT) in the concentration range of 0.01–10.0%. The control consisted of HaCaT cells not exposed to any extract, for which viability was assumed to be 100%. Three independent experiments were performed, in which each concentration was tested in four replicates. **** *p* < 0.0001 versus the control.

The analyses performed using resazurin (AB assay) indicated that the effect of feverfew extracts on the viability of fibroblasts and keratinocytes depended on their concentration and on the part of the plant. The most beneficial effect on BJ cells was observed for TFE and THE, with only the highest concentration analyzed (10.0%) causing a cytotoxic effect. In the case of TLE, concentrations of both 5.0% and 10.0% resulted in a decrease in the viability of these cells. Lower concentrations of all extracts tested resulted in a statistically significant increase in the metabolic activity of fibroblasts (Figure 2). Analyses performed on keratinocytes (Figure 3) showed similar effects, with the most favorable effect obtained in the case of TFE. For all extracts tested, the most beneficial concentrations were those not exceeding 1.0%.

**Figure 4:**
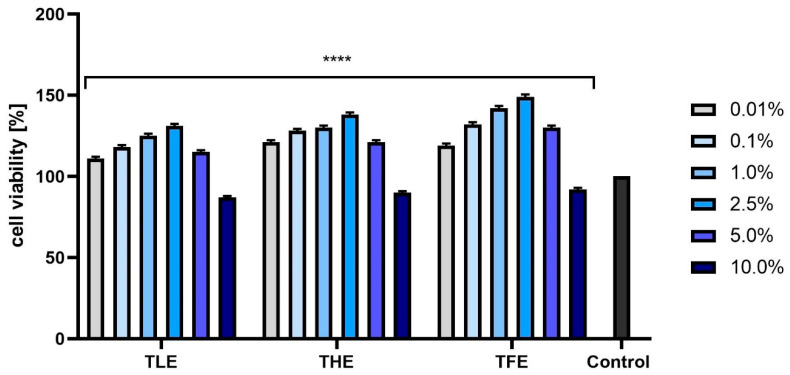
Effect of extracts from *Tanacetum parthenium* leaves (TLE), herb (THE), and flower heads (TFE) on neutral red dye uptake in fibroblasts (BJ) in the concentration range of 0.01–10.0%. The control consisted of BJ cells not exposed to any extract, for which viability was assumed to be 100%. Three independent experiments were performed, in which each concentration was tested in four replicates. **** *p* < 0.0001 versus the control.

**Figure 5:**
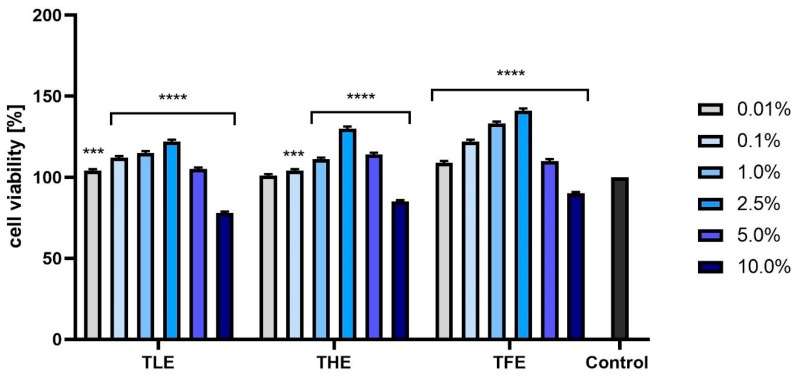
Effect of extracts from *Tanacetum parthenium* leaves (TLE), herb (THE), and flower heads (TFE) on neutral red dye uptake in keratinocytes (HaCaT) in the concentration range of 0.01–10.0%. The control consisted of HaCaT cells not exposed to any extract, for which viability was assumed to be 100%. Three independent experiments were performed, in which each concentration was tested in four replicates. **** *p* < 0.0001, *** *p* ≤ 0.001 versus the control.

In the case of the NR assay, the feverfew extracts exerted a very similar effect on both types of skin cells. For all types of extracts, the viability of these cells increased with the concentration of the extract (up to 2.5%). Similarly to the AB test, a 10% concentration of TLE, THE, and TFE produced cytotoxic effects (Figure 4 and Figure 5). To sum up, both tests showed that TFE has the strongest pro-proliferative effect on fibroblasts and keratinocytes, especially at concentrations of 1.0% and 2.5%. The other two types of extracts can also increase the viability of these cells but to a slightly smaller extent.

### 2.5. Antioxidant Activity

The hydroethanolic extracts from various parts of feverfew were examined using DPPH free radical-scavenging methods and the ferric-reducing antioxidant power assay (FRAP) (Table 5). The extract obtained from flowers showed the highest antioxidant activity in these two in vitro tests (84.09% and 1.54 mmol/L, respectively).

**Figure 6:**
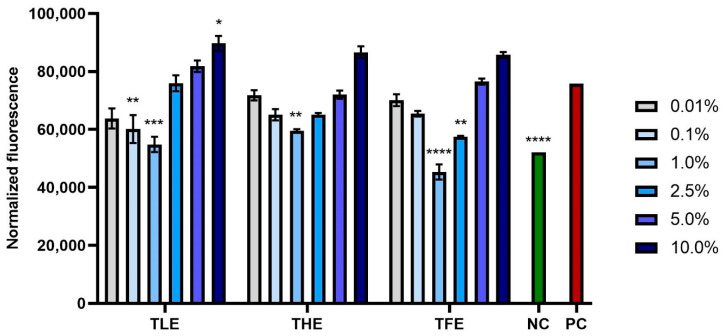
Effect of extracts from *Tanacetum parthenium* leaves (TLE), herb (THE), and flower heads (TFE) on the intracellular level of reactive oxygen species in fibroblasts (BJ) treated with 500 µM of H_2_O_2_. The results show the fluorescence of 2′,7′-dichlorofluorescein (DCF) in cells previously treated for 24 h with extracts from various parts of *Tanacetum parthenium* in the concentration range of 0.01–10.00%. The negative control (NC) was BJ cells treated with neither 500 µM of H_2_O_2_ nor extracts, and the positive control (PC) was cells treated only with 500 µM H_2_O_2_. Data represent the mean ± SD of three independent experiments, with each sample analyzed in four replicates. **** *p* < 0.0001, *** *p* = 0.0003, ** *p* < 0.01, * *p* = 0.0244 compared to positive control.

**Figure 7:**
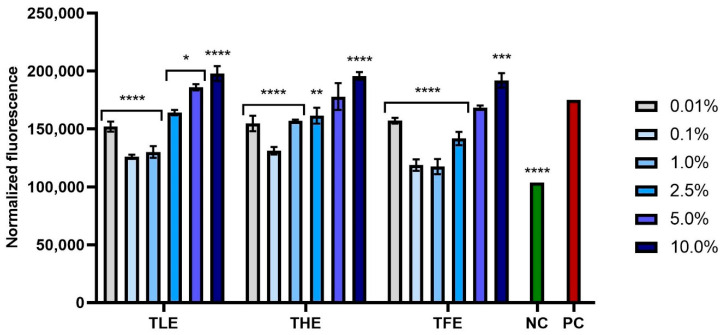
Effect of extracts from *Tanacetum parthenium* leaves (TLE), herb (THE), and flower heads (TFE) on the intracellular level of reactive oxygen species in keratinocytes (HaCaT) treated with 500 µM of H_2_O_2_. The results show the fluorescence of 2′,7′-dichlorofluorescein (DCF) in cells previously treated for 24 h with extracts from various parts of *Tanacetum parthenium* in the concentration range of 0.01–10.00%. The negative control (NC) was HaCaT cells treated with neither 500 µM of H_2_O_2_ nor extracts, and the positive control (PC) was cells treated only with 500 µM of H_2_O_2_. Data represent the mean ± SD of three independent experiments, with each sample analyzed in four replicates. **** *p* < 0.0001, *** *p* = 0.0004, ** *p* = 0.0034, * *p* < 0.05 compared to positive control.

This study also assessed the impact of feverfew extracts at various concentrations on ROS levels following exposure to hydrogen peroxide as a pro-oxidant agent. Exposing fibroblasts and keratinocytes to 500 µM of H_2_O_2_ caused a significant increase in the level of free radicals in both types of cells (positive controls). Differences in antioxidant activity were noted between extracts from different parts of the plant and between the concentrations used. The extract causing the greatest reduction in the level of ROS in both cell types was TFE, which is consistent with the results obtained in the assay using DPPH radicals. The optimal concentration of this extract was 1.0%. All extracts tested (TLE, THE, and TFE) at concentrations above 5.0% caused a significant increase in fluorescence in both BJ and HaCaT cells, which indicates the induction of oxidative stress. Taking into account the results obtained for all types of extracts, it can be concluded that *T. parthenium* extracts at a concentration below 2.5% have the strongest antioxidant properties, causing the greatest reduction in oxidative stress (Figure 6 and Figure 7). Although these extracts significantly reduce the level of ROS in cells exposed to H_2_O_2_, they are not able to completely eliminate the unfavorable effect of this pro-oxidant compound on skin cells, as the fluorescence in cells not exposed to H_2_O_2_ (negative control) is lower than in cells pre-treated with extracts and then treated with H_2_O_2_.

### 2.6. Antimicrobial Activity

In this study, the antimicrobial properties of extracts from different parts of the feverfew plant against Gram-positive bacteria, Gram-negative bacteria, and fungi were analyzed. The results indicated that extracts from the herb had the lowest overall MIC values, making them the most effective at inhibiting microbial growth. This finding was consistent across all types of microorganisms tested. There were no statistically significant differences between TLE, THE, and TFE in minimum inhibitory concentrations (MICs) against the bacterial strains tested (*p*-value = 0.444). The herb extract consistently showed the lowest MIC values across different types of microorganisms, confirming its superior antimicrobial efficacy. Specifically, among the microbes tested, MIC values for THE (0.25 mg/mL) were lowest against *Candida albicans* and *Streptococcus pneumoniae*. Among Gram-negative bacteria, TLE was more effective. The lowest MIC values for Gram-negative bacteria were observed for *Proteus mirabilis* (0.25 mg/mL) treated with TLE. The analysis indicates that the MIC values for all plant parts were lowest against fungi, suggesting greater sensitivity to the extracts. MIC values against Gram-negative bacteria were lower compared to Gram-positive bacteria, for which the MIC values were the highest. This means that Gram-positive bacteria require higher concentrations of extracts to inhibit growth than Gram-negative bacteria and fungi (Table 6).

The anti-biofilm activity of extracts from different parts of the feverfew herb was tested against such microorganisms as Gram-positive bacteria, Gram-negative bacteria, and fungi. The Kruskal–Wallis test indicated that there was no statistically significant difference in the percentage of biofilm inhibition between extracts obtained from different parts of the plants (*p*-value = 0.436). This result was consistent across all groups of microorganisms. Further analysis using the Kruskal–Wallis test revealed that both the extract concentration and the type of microorganism significantly influenced biofilm inhibition (*p* < 0.05).

The highest mean biofilm inhibition was observed at concentrations of 4 mg/mL (56.95%) and 2 mg/mL (53.11%). This indicates that the effectiveness of the extract increases at higher concentrations, which may potentially have stronger anti-biofilm effects. The effectiveness of biofilm inhibition varied depending on the type of microorganism. The greatest sensitivity to the anti-biofilm action of the extracts was shown by *Proteus mirabilis* (60.86%) and *Candida albicans* (60.36%). High inhibition was also noted for *Pseudomonas aeruginosa* (56.54%) and *Streptococcus pyogenes* (50.60%). In contrast, *Staphylococcus epidermidis* showed no inhibition. Regarding the part of the plant, the highest anti-biofilm properties were exhibited by extracts from flowers (42.74%), surpassing the effectiveness of extracts from the herb (39.98%) and leaves (35.73%) (Table 7).

## 3. Discussion

The content of bioactive compounds in plant materials may differ depending on geographic location, climate, soil type, stage of growth, or the part of the plant the samples are obtained from. Exogenous and endogenous factors can affect the content of bioactive compounds in the plant [10]. *Tanacetum parthenium* is rich in active constituents such as sesquiterpene lactones, polyphenolic compounds, and volatile oils, among others [11].

In the present study, a total of 61, 68, and 83 components were detected in the essential oils of the leaves, herbs, and flower heads of feverfew plants, representing 98.37%, 97.53%, and 99.37%, respectively, of the total composition. The major compounds of the EO from flower heads were chrysanthenone (14.08%), α-thujone (9.60%), tetradecane (6.96%), camphor (6.30%), and α-cadinol (5.84%). The results revealed similar content of the dominant volatile compounds (camphor, trans-chrysanthenyl acetate, and camphene) present in the essential oils of the leaves (40.14%, 22.17%, and 7.12%, respectively) and herb (38.96%, 25.26%, and 6.57%, respectively). Our findings are consistent with those of other authors. According to the literature, the main volatile compounds identified in EO of *T. parthenium* from different geographic regions were also camphor, camphene, and chrysanthenyl acetate, but the amounts were highly varied. Very similar quantitative and qualitative results to those obtained in this study for the composition of *T. parthenium* EO isolated from stems and flowers were reported by researchers from Turkey, who also found that the main volatile compounds were camphor (49%), trans-chrysanthenyl acetate (22.1%), and camphene (9.4%) [6]. Similarly, the dominant compounds in EO obtained from the flowering aerial parts of *T. parthenium* L. in South Bulgaria were also camphor (45.47%), trans-chrysanthenyl acetate (21.65%), and camphene (9.48%), in addition to *cis*-isogeraniol (5.42%) [12]. Camphor, chrysanthenyl acetate, and camphene were also dominant in EO from *T. parthenium* grown in Egypt [13], while camphor and camphene were the main compounds found in EO obtained from *T. parthenium* grown in Kosovo [14] and Tajikistan [15]. Another study evaluating *T. parthenium* EO of Danish origin found that the major components were camphor, *trans*-chrysanthenyl acetate, and trans-chrysanthenol, at levels of 26.7%, 15.7%, and 7.7%, respectively [16]. EO extracted from the aerial parts of *T. parthenium* in Iran had a slightly different composition and content of the main components. The researchers reported the highest content of camphor (18.94%), bornyl acetate (18.35%), camphene (13.74%), bornyl isovalerate (3.15%), borneol (10.93%), juniper camphor (6.23%) and β-eudesmol (2.65%) at the flowering stage of the plant [17]. Other researchers from Iran showed differences in the composition of EO depending on the part of the plant from which it was obtained: EO from the leaves contained a high amount of camphor (56.4%); EO from the stem mainly contained camphor (26.0%), trans-beta-ocimene (23.6%), and germacrene-d (15.0%); and the major constituents of the root EO were alpha-pinene (50.0%), trans-beta-farnesene (13.8%), and bicyclogermacrene (11.0%) [18]. The differences in the chemical components of feverfew essential oil obtained from different geographical regions are related to exogenous and endogenous factors, such as climate, soil conditions, growing stage, and cultivation method, as well as the collection time, part of plant or methods, and equipment used in the analysis. These factors may affect the volatile organic composition and amounts of terpene in the essential oil [6,12].

To our knowledge, apart from the determination of volatile compounds, data on the chemical composition of *T. parthenium* extracts from different parts of the plant are limited. To fill this gap in the literature, this study presents an analysis of the content of total polyphenols, total flavonoids, total phenolic acids, condensed tannins, and terpenoids conducted using spectrophotometric methods, as well as a qualitative and quantitative analysis of flavonoids and phenolic acids conducted using HPLC.

The spectrophotometric analyses showed that TFE had the highest content of total polyphenols (67.41 mg/mL), flavonoids (19.33 mg/mL), and phenolic acids (5.10 mg/mL), while THE had the highest content of condensed tannins (22.89 mg/mL) and terpenoids (54.41 mg/mL). In a study by Hordiei et al. [11], quantitative determinations carried out via spectrophotometry revealed that the flavonoid content in feverfew herb samples ranged from 0.79% to 2.65%, while the amounts of hydroxycinnamic acids in the samples ranged from 3.34% to 6.47%, depending on the cultivation location. Wu et al. [19] determined the TPC of feverfew extract to be 21.21 μg of GAE/mg dry matter.

The analyses carried out using HPLC showed that the major polyphenol compounds present in the hydroethanolic extracts of *T. parthenium* leaves, herbs, and flower heads were 3,5-dicaffeoyl-quinic acid, chlorogenic acid, and 3,4-dicaffeoyl-quinic acid. Another study using GC–MS and HPLC–UV to determine the bioactive constituents of feverfew powder extracted with 80% alcohol showed that it contained camphor, parthenolide, luteolin, and apigenin [19]. Other researchers showed the presence of seven polyphenols, i.e., ferulic acid, apigenin, luteolin-7-O-glucoside, luteolin, chrysosplenol, kaempferol, and santin, in methanolic extracts from *T. parthenium* [20]. In another study, HPLC analysis revealed the presence of three flavonols (quercetin, kaempferol-3-rutinoside, and o-methylated flavonol santin), one flavone (apigenin), and six hydroxycinnamic acids (chlorogenic, 4-O-caffeoyl-quinic, 3,4-dicaffeoyl-quinic, 3,5-dicaffeoyl-quinic, 4,5-dicaffeoyl-quinic, and neochlorogenic acid) [11]. Silveira de Almeida et al. [21] separated the flavonol santin and the flavone apigenin. Végh et al. [22] detected three other lipophilic flavonoids: sudachitin, aceronin, and nevadensin. Another study showed that the lipophilic flavonoids present in the leaves and flowers of *T. parthenium* are methyl ethers of the flavonols 6-hydroxykaempferol and quercetagetin [23]. Wu et al. [24] detected the presence of 3,5-, 4,5- and 3,4-di-O-caffeoylquinic acids with potent DPPH scavenging activity. Determining the phenolic compounds in plant raw material is crucial due to their notable role as antioxidant free radical scavengers [25]. Previous research results on radical scavenging activity demonstrated that the feverfew herb samples with the highest content of flavonoids and phenolic acids exhibited the most pronounced antioxidant activity [20].

It is worth mentioning here that certain phenolic compounds can exhibit strong antioxidant activity even at low concentrations, while others, despite being present at high concentrations, may have limited or no antioxidant activity [11]. For example, the phenolic compounds vanillic acid, p-hydroxybenzaldehyde, and p-coumaric acid did not react against the DPPH radical [25]. It is also interesting to note that the antioxidant activity of a sample can be influenced by other non-phenolic compounds present in the extract, which can significantly contribute to antioxidant activity, leading to discrepancies between total phenolic content and antioxidant capacity [11]. Natural antioxidants such as phenolic acids, flavonoids, and tannins are found in different plant parts (e.g., fruits, flowers, and leaves) and have been reported to have a number of biological effects, including antioxidant activity [26]. The ability to scavenge free radicals is a significant parameter for the quality of plant extracts and their potential use in prevention and therapy. The in vitro tests (DPPH and FRAP) carried out in the present study are valued as simple, fast, and cost-effective tools for measuring the antioxidant activity of plant extracts [27]. Therefore, they can provide important information regarding the potential use of the feverfew extracts with the highest antioxidant capacity. Generally, TFE (84.9% DPPH and 1.54 mmol/L of FRAP) exhibited higher antioxidant activity than THE (69.54% DPPH and 1.43 mmol/L of FRAP) and TLE (64.73% DPPH and 1.34 mmol/L of FRAP). Other researchers determined the DPPH radical scavenging capacity of feverfew methanolic extracts at a level of 88.6% [20]. Prashanth et al. [28] evaluated the ability of ethanolic and aqueous extracts from *T. parthenium* leaves to scavenge DPPH (63.53% and 67.90%, respectively), ABTS (86.011% and 86.55%), and superoxide anion (59.16 µg/mL and 68.88 µg/mL) radicals, as well as their ferric-reducing antioxidant power (0.135 µg/mL and 0.141 µg/mL) and total antioxidant capacity (2.22 mM/L and 1.316 mM/L, respectively). Hordiei et al. [11] investigated the antioxidant activity of feverfew herb using the ABTS radical cation scavenging assay. Radical scavenging activity ranged from 86.49 ± 1.07 µmol/g to 127.89 ± 1.04 µmol/g for samples obtained from different regions of Ukraine [11]. Wu et al. [19] determined the antioxidant activity of a feverfew alcoholic extract based on its free radical scavenging activity against the DPPH radical (84.4%) and its Fe^2+^ chelating capacity (53.1%). Herrera-Calderon et al. [29] reported the antioxidant activity of an ethanolic extract of *T. parthenium* leaves and flowers against DPPH to be IC_50_ 452.10 μg/mL and IC_50_ 270.70 μg/mL, respectively.

The ability to protect cells against the negative effects of numerous chemical compounds and factors responsible for oxidative stress is extremely important for the maintenance of normal cell morphology and activity. The differences in the influence of the extracts on the metabolic activity and viability of skin cells in vitro are undoubtedly due to differences in the content of biologically active compounds in different parts of *T. parthenium* L. [1]. The results presented here clearly indicate that the effect of all extracts tested is strictly dose-dependent, so it is extremely important to select concentrations that will ensure the desired effect. Many mechanisms of action may be responsible for the positive effect of the extracts. Their protective effect may be the result of increasing the level of endogenous antioxidants in the skin cells and inducing various DNA repair mechanisms. This action may be based on the activation of the NF-E2-related factor-2 (Nrf2)—antioxidant response element (ARE) pathway [30]. Piela-Smith and Liu [31] also indicated that feverfew extracts may inhibit the expression of intercellular adhesion molecule-1 (ICAM-1) induced by the cytokines IL-1, interferon-gamma, and TNF-alpha. The cellular protective effect may also be due to the well-documented anti-inflammatory effects of feverfew [1,32]. Sur et al. indicated that parthenolide-depleted extract of *T. parthenium* L. can inhibit the activity of pro-inflammatory enzymes such as 5-lipoxygenase, phosphodiesterase-3, and phosphodiesterase-4. This extract also showed the ability to inhibit the release of pro-inflammatory mediators nitric oxide, PGE(2), IFN-gamma, TNF-alpha, IL-2, and IL-4 [8]. The cytotoxic effect of higher concentrations of feverfew extracts may be linked to the disruption of DNA replication in the cell lines and inhibition of thymidine in DNA. This may be due to the highly reactive lactone ring and the epoxy and methylene groups present in the parthenolide molecule in extracts from this plant [1]. These extracts may also increase intracellular oxidative stress and result in mitochondrial dysfunction and intracellular thiol depletion [1,33,34].

Several recent studies have provided new evidence for the antibacterial activity of *T. parthenium* essential oil [6,17,18]. To the best of our knowledge, no previous research has investigated the antimicrobial properties of feverfew extracts. The antimicrobial activity of hydroethanolic extracts obtained from different parts of the plant was tested against 12 different microorganisms, including Staphylococcus aureus, *Streptococcus epidermidis*, and *Pseudomonas aeruginosa* as the three major bacterial strains causing skin diseases or wound infections. This study represents the first comprehensive investigation into the antimicrobial properties of hydroethanolic extracts from different parts of *Tanacetum parthenium*, contrasting with prior research that primarily focused on essential oils and individual bioactive compounds [6,17,18,35,36,37,38]. Our findings underscore the potential of feverfew extracts as effective antimicrobial agents, providing novel insights into their application against a broad spectrum of microorganisms. The superior efficacy of herb extracts observed in this study highlights the presence of a synergistic combination of bioactive compounds. This finding aligns with the established antimicrobial activities of essential oils, which vary depending on the plant part and developmental stage, affecting their chemical composition and potency. Compounds such as camphor and camphene, known for their antimicrobial properties, likely contribute to these effects, as suggested by previous studies on feverfew essential oils [17]. These two compounds, together with trans-chrysanthenyl acetate, are the predominant substances in the essential oils from the feverfew herb and leaves tested in the present study. Moreover, some studies have reported that polyphenols, including flavonoids, phenolic acids, and tannins, show good antimicrobial effects and inhibit the growth of diverse microbes, including Gram-positive and Gram-negative bacteria and fungi [39,40]. The presence of triterpenoids such as oleanolic acid, betulinic acid, and ursolic acid, known for their antimicrobial properties against pathogens such as *Mycobacterium tuberculosis*, supports the antimicrobial potential of feverfew extracts [35]. Additionally, the ability of feverfew extracts to inhibit biofilm formation, particularly at higher concentrations, further underscores their potential in managing chronic infections. Parthenolide, a major bioactive component of feverfew, has shown significant anti-quorum sensing and anti-biofilm activities, which may explain the efficacy observed against biofilm-forming pathogens such as *Pseudomonas aeruginosa* [37]. This makes feverfew a promising candidate for the development of therapies targeting biofilm-associated infections. The protective effects of parthenolide against gentamicin-induced nephrotoxicity and oxidative stress further suggest its therapeutic potential in reducing the toxicity of antimicrobial treatments [38]. Moreover, the inhibition of cytochrome P450 3A4 (CYP3A4) activity by feverfew extracts suggests potential therapeutic applications through the modulation of drug-metabolizing enzymes [36]. This inhibition could enhance the effectiveness of other antimicrobial agents used in combination with feverfew extracts. The findings from this study suggest several practical applications for feverfew extracts in clinical settings. The broad-spectrum antimicrobial activity, particularly against pathogens such as *Candida albicans* and *Proteus mirabilis*, indicates potential use in treating fungal and bacterial infections that are resistant to conventional treatments. Moreover, the anti-biofilm properties of feverfew extracts could be harnessed to develop new strategies for preventing and treating biofilm-related infections.

## 4. Materials and Methods

### 4.1. Chemicals

The reagents used were deionized water, ethanol, butanol (POCH, Gliwice, Poland), Folin–Ciocalteu (F-C) reagent, sodium carbonate, sodium nitrite, sodium hydroxide, aluminium chloride (Chempur, Piekary Śląskie, Poland), sodium acetate, ferric chloride (Warchem, Zakręt, Poland), gallic acid, 2,4,6-tripyridyl-s-triazine (TPTZ), 1,1-diphenyl-2-picrylhydrazyl (DPPH), hydrochloric acid, Arnova reagent, caffeic acid, delphinidin, catechin, sulfuric acid, chloroform, linalool, *Trolox* (6-hydroxy-2,5,7,8-tetramethylchroman-2-carboxylic acid), brain heart infusion broth (BHI, Graso, Owidz, Poland), and antibiotics —streptomycin, erythromycin, and fluconazole (Sigma-Aldrich, Poznań, Poland). The standards for HPLC analysis were apigenin, quercetin, santin, kaempferol-3-rutinoside, chlorogenic acid, neochlorogenic acid, 4-O-caffeoyl-quinic acid, 3,4-dicaffeoyl-quinic acid, 3,5-dicaffeoyl-quinic acid, 4,5-dicaffeoyl-quinic acid, and ellagic acid (Sigma-Aldrich, Saint Louis, MO, USA). All standards, reagents, and solvents were of analytical grade. The reagents used in the cell culture were DMEM (Dulbecco’s Modified Eagle’s Medium; Biological Industries, Cromwell, CO, USA), antibiotics (100 U/mL penicillin and 1000 μg/mL streptomycin; Thermo Fisher Scientific, Waltham, MA, USA), foetal bovine serum (FBS; Gibco, Waltham, MA, USA), phosphate-buffered saline (PBS, Genos, Łódź, Poland), 2′,7′-dichlorodihydrofluorescein diacetate (H2DCFDA), trypsin-EDTA solution, resazurin sodium salt, neutral red dye (Merck KGaA, Darmstadt, Germany), hydrogen peroxide, ethanol, and acetic acid (CH_3_COOH; Warchem, Zakręt, Poland).

### 4.2. Sample Collection and Preparation

**Figure 8:**
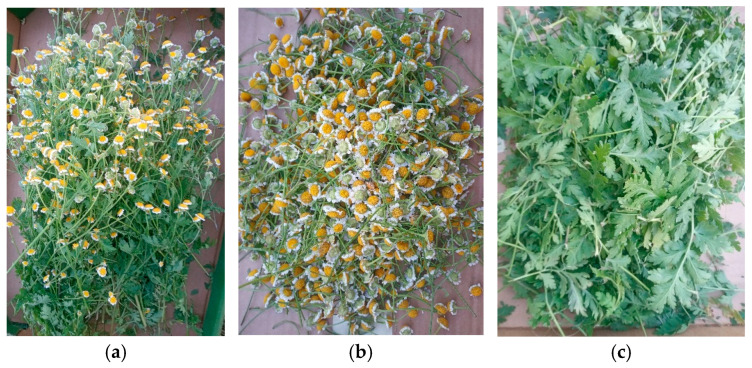
Parts of feverfew used in the study: (**a**) aerial parts, (**b**) flower heads, (**c**) leaves.

The feverfew plants for the study were collected in full bloom in June 2023 from the collection of the Garden of Cosmetic Plants and Raw Materials, Research and Science Innovation Centre) in the village of Wola Zadybska (51°45′ N 21°51′ E), near Lublin, Poland. The plants were cut at a height of 20 cm above the ground. Some of the plants were prepared whole (a) for drying immediately after collection, while the flower heads (b) and leaves (c) were separated by hand from the remaining plants (Figure 8). All of the materials were dried in a forced-air laboratory oven at 35 °C. The plant structure was determined as the proportion of flower heads and leaves in the weight of the aerial parts, as well as the loss of weight after the individual plant parts were dried. The plant material was authenticated by A. Kiełtyka-Dadasiewicz. Voucher specimens of *T. parthenium* were deposited in the Research and Science Innovation Centre (No. 16-18/2023). The dried material was ground to a powder in an electric grinder (MF 10 basic, IKA-werke, Staufen, Germany).

### 4.3. Isolation and Analysis of Essential Oils

The dried samples (15 g) of each part of the plant (separately) were placed in round-bottom flasks with a capacity of 1000 mL. Each flask was filled with 500 mL of distilled water, and the contents were vapour-distilled for a period of 24 h using a Clevenger apparatus. The extracted oils were dried and stored in the dark in tightly sealed vials at 4 °C until analysis (GC/MS). The distillation process was carried out in triplicate for each part of the plant. GC/MS qualitative analysis of the isolated oils was carried out using a Hewlett Packard (HP 6890) gas chromatograph equipped with an HP-5MS capillary column (30 m × 0.25 mm; film thickness 0.25 μm) and an HP 5973 mass selective detector. The carrier gas was helium, with a flow rate of 1 mL/min. Samples in the amount of 2 μL (40 mg oil dissolved in 1.5 mL methylene chloride) were injected in split mode at a 5:1 ratio. The injector and transfer line temperature was 280 °C, and the ion source temperature was 230 °C. The initial column temperature was held at 40 °C for 5 min, then increased to 60 °C at a rate of 3 °C/min, then to 230 °C at 6 °C/min (held for 10 min), and then to a final temperature of 280 °C at 3 °C/min (held for 30 min). The total run time for a single sample was 76 min. The composition of the essential oils was determined by comparing their retention indices (RI) with those given in the NIST Chemistry Web-Book and the literature [41]. RIs were calculated using a mixture of homologous series of n-alkanes (C7-C40, Supelco, Bellefonte, PA, USA) in the same chromatographic conditions as for the essential oils. Further identification of individual constituents was carried out by comparing their mass spectra with those stored in the NBS75K and NIST 2002 mass spectral libraries. Relative percentages of essential oil constituents were determined on the basis of total peak areas using the chromatograph software 4.10.

### 4.4. Preparation of Feverfew Extracts

For the hydroethanolic extracts from the herb (THE), flower heads (TFE), and leaves (TLE) of feverfew, 2 g of powdered sample was sonificated twice with 60 mL of an ethanol-water mixture (50:50, *v*/*v*) for 60 min using an ultrasonic bath (40 kHz, 2 × 320 W) (Polsonic 5, Warsaw, Poland). Then, the extracts were filtered using Whatman paper.

### 4.5. Total Phenolic Content of Feverfew Extracts

Total phenolic content was determined using the Folin–Ciocalteu method [42]. Standard solutions of gallic acid with a known concentration were used to prepare a calibration graph. The test was performed in triplicate. The results were expressed as mg of gallic acid equivalent (GAE) per mL of each extract. The absorbance was assessed spectrophotometrically at 765 nm using the UV-1900i UV-Vis spectrophotometer (Shimadzu, Kyoto, Japan).

### 4.6. Total Flavonoid Content of Feverfew Extracts

Total flavonoid content was determined according to Kim et al. [43]. Standard solutions of catechin with a known concentration were used to prepare a calibration graph. The test was performed in triplicate. The results were expressed as mg of catechin equivalent (CE) per mL of each extract. The absorbance was assessed spectrophotometrically at 510 nm using the UV-1900i UV-Vis spectrophotometer (Shimadzu, Kyoto, Japan).

### 4.7. Total Phenolic Acid Content of Feverfew Extracts

Total phenolic acid content was estimated according to Jain et al. [44]. Standard solutions of caffeic acid with a known concentration were used to prepare a calibration graph. The test was performed in triplicate. The results were expressed as mg of caffeic acid equivalents (CAE) per mL of each extract. The absorbance was assessed spectrophotometrically at 490 nm using the UV-1900i UV-Vis spectrophotometer (Shimadzu, Kyoto, Japan).

### 4.8. Condensed Tannin Content of Feverfew Extracts

Condensed tannin content was assessed according to Tlili et al. [45]. Standard solutions of delphinidin with a known concentration were used to prepare a calibration graph. The test was carried out in triplicate. The results were expressed as mg of delphinidin equivalents (DpE) per mL of each extract. The absorbance was estimated spectrophotometrically at 550 nm using the UV-1900i UV-Vis spectrophotometer (Shimadzu, Kyoto, Japan).

### 4.9. Terpenoid Content of Feverfew Extracts

Terpenoid content was measured according to Ghorai et al. [46]. Standard solutions of linalool with a known concentration were used to prepare a calibration graph. The results were expressed as linalool (mg LL per mL of extract). Analyses were carried out in three independent experiments. The terpenoid content was assessed spectrophotometrically at 538 nm using a UV-1900i UV-Vis spectrophotometer (Shimadzu, Kyoto, Japan).

### 4.10. Determination of Phenolics and Flavonoids of Feverfew Extracts by HPLC

The Shimadzu HPLC-DAD System (Shimadzu, Kyoto, Japan), consisting of a degasser (DGU-14A), dual plunger pump (LC-10AT VP), automatic injector (SIL-10ADvp), column oven (CTO-10A VP), diode array detector (Prominence SPD-M20A), and system controller (SCL-10VP), was used to analyze phenolic compounds. Chromatographic separation was carried out in a Bionacom STR column (2.5 μm, 3 mm × 100 mm) using a gradient program with two solvent systems. The mobile phase consisted of eluent A (0.05% trifluoroacetic acid) and eluent B (acetonitrile). The gradient program was set as follows: 0–5 min—12% B, 5–50 min—12–30% B, 50–51 min—30–90% B, 51–56 min—90% B, and 57 min—12% B, with a flow rate of 0.5 mL/min. The injection volume was 10 μL. Peaks were identified using the retention time of the analyte, a reference compound, and the UV absorption spectrum. Calibration curves of the reference compounds were prepared. The content of phenolic acids was assessed at a wavelength of 325 nm, and the flavonoid content was assessed at 350 nm.

### 4.11. Cell Culture

Two cell lines were used in the tests assessing the cytotoxicity of the extracts and their effect on the intracellular ROS level: normal human HaCaT keratinocytes (CLS Cell Lines Service GmbH, Eppelheim, Germany) and BJ fibroblasts (American Type Culture Collection, Manassas, VA, USA). Cells were cultured in 75 cm^2^ culture flasks (Googlab Scientific, Rokocin, Poland) in an incubator at 37 °C and an atmosphere containing 95.0% air and 5.0% carbon dioxide. Cells were cultured in DMEM (Dulbecco’s Modified Eagle’s Medium, Biological Industries, Cromwell, CO, USA), which was additionally supplemented with sodium pyruvate, L-glutamine, glucose (4.5 g/L), and 10.0% foetal bovine serum (Gibco, Waltham, MA, USA). To avoid contamination, 1.0% antibiotics (100 U/mL of penicillin and 1000 μg/mL of streptomycin, Thermo Fisher Scientific, Waltham, MA, USA) were also added to the medium. Cells were passaged using trypsinization after reaching approximately 70–80.0% confluence.

### 4.12. Cytotoxicity Analysis—Alamar Blue (AB) and Neutral Red (NR) Uptake Assays

The cytotoxicity of the extracts was assessed using resazurin sodium salt (Alamar blue assay, Merck KGaA, Darmstadt, Germany) and neutral red dye (neutral red assay, Merck KGaA, Darmstadt, Germany) according to the methodology previously described by Ziemlewska et al. [47]. For this purpose, fibroblasts and keratinocytes were seeded separately in 96-well sterile flat-bottom plates (Googlab Scientific, Rokocin, Poland). Black plates were used for the AB test and standard transparent ones for the NR test. After the cultured cells attached to the bottom of the plates (after about 24 h), the DMEM medium was aspirated and replaced with individual dilutions of the tested extracts. Cells were treated with TFE, TLE, and THE extracts in the concentration range of 0.01–10.0% for a period of 24 h. Then, for the resazurin test, the extract solutions were aspirated and replaced with a 60 µM resazurin solution, followed by incubation for 2 h at 37 °C. For the NR test, after aspirating the extracts, the cells were treated with a neutral red dye (40 µg/mL) for 2 h at 37 °C and then washed three times with phosphate-buffered saline (PBS, Genos, Łódź, Poland) and treated with a decolourizing buffer (C_2_H_5_OH/CH_3_COOH/H_2_O, 50.0%/1.0%/49.0%) for 15 min. Then, for the AB assay, the fluorescence of cells in individual wells was measured at λ = 570 nm using a microplate reader (ThermoFisher Scientific, Waltham, MA, USA), while in the NR assay, the absorbance was determined at λ = 540 nm. The control sample consisted of cells (HaCaT and BJ separately) cultured in DMEM medium without the addition of *T. parthenium* extracts, for which the viability was assumed to be 100%. Separate controls were performed for the AB and NR tests. Cytotoxicity analyses were carried out in three independent experiments in which each concentration of extracts was tested four times.

### 4.13. Antioxidant Activity Evaluation of Feverfew Extracts

#### 4.13.1. DPPH Scavenging

The antioxidant properties of the extracts were determined using the DPPH free radical scavenging assay [48]. Briefly, 50 μL of extract or ethanol (as a blank) was mixed with 0.5 mL of DPPH solution and left for 20 min in the dark. Inhibition of the DPPH radical by the sample was calculated using the following formula: DPPH scavenging activity (%) = [A_0_ − A_1_/A_0_] × 100, where A_0_ is the absorbance of the control, and A_1_ is the absorbance of the sample. All experiments were performed in triplicate, and the results were reported as means ± SD of triplicates. Free radical scavenging activity is expressed as the percentage of DPPH decrease. The absorbance was assessed at 517 nm using the UV-1900i UV-Vis spectrophotometer (Shimadzu, Kyoto, Japan).

#### 4.13.2. The Ferric-Reducing Antioxidant Power (FRAP Assay)

The FRAP assay was performed using the method proposed by Benzie and Strain [49]. Briefly, the FRAP reagent (prepared by mixing acetate buffer (300 mM, pH 3.6), a solution of 10 mM of TPTZ in 40 mM of HCl, and 20 mM of FeCl_3_ at 10:1:1 (*v*/*v*/*v*)) was mixed with a defined concentration of the plant extracts and incubated at 37 °C for 4 min. Standard curve was construct using different concentrations of trolox. The obtained results were expressed as mmol of Trolox equivalent (TE) per 1 L of each extract. Each sample was prepared in triplicate. The absorbance was assessed at 593 nm using the UV-1900i UV-Vis spectrophotometer (Shimadzu, Kyoto, Japan).

#### 4.13.3. Determination of Intracellular Level of Reactive Oxygen Species (ROS)

The effect of TLE, THE, and TFE extracts on the intracellular ROS level in fibroblasts and keratinocytes was assessed using the fluorogenic probe 2′,7′-dichlorodihydrofluorescein diacetate (H_2_DCFDA, Merck KGaA, Darmstadt, Germany) according to a previously described procedure [50]. Briefly, both cell lines were seeded in 96-well plates at a density of 1 × 10^4^ cells per well. After the cells had attached to the bottom (about 20 h), the DMEM medium (Dulbecco’s Modified Eagle Medium, Biological Industries, Cromwell, CO, USA) was aspirated and replaced with individual dilutions of the extracts (in the concentration range of 0.01–10.0%) prepared in the culture medium. Fibroblasts and keratinocytes were incubated with the extracts for 24 h in an incubator. Then, the individual extract solutions were aspirated from the wells and replaced with a solution of 10 μM of H_2_DCFDA dissolved in serum-free DMEM medium. Hydrogen peroxide (H_2_O_2_) solution was immediately added to the extract wells, reaching a final concentration of 500 μM in each well. Positive controls were cells treated with 500 µM of H_2_O_2_ but without prior treatment with extracts. The negative control consisted of cells cultured in DMEM medium (without exposure to extracts and H_2_O_2_). After 60 min of incubation, the fluorescence was measured in each well at an excitation wavelength of λ = 485 nm and an emission wavelength of λ = 530 nm using a microplate reader (FilterMax F5, Thermo Fisher Scientific, Waltham, MA, USA). Three independent experiments were carried out, in which each extract concentration was evaluated in four replicates.

### 4.14. Antimicrobial Activity Evaluation of Feverfew Extracts

#### 4.14.1. Microorganisms

The antimicrobial properties of tested extracts were assessed against 12 microorganisms, including Gram-positive bacteria, i.e., *Staphylococcus aureus* PCM 2054, *Staphylococcus epidermidis* PCM 2118, *Streptococcus agalactiae* PCM 2683, *Streptococcus pyogenes* PCM 2855, *Streptococcus mutans* PCM 2502, *Streptococcus pneumoniae* PCM 2589, and *Enterococcus faecalis* PCM 2673; Gram-negative bacteria, i.e., *Pseudomonas aeruginosa* PAO1 (biofilm model strain), *Escherichia coli* PCM 2057, *Proteus mirabilis* S1959, and *Shigella sonnei* PCM 2336; and one fungal strain, *Candida albicans* ATTC 10231. All strains were sourced from the Polish Collection of Microorganisms in Wroclaw, Poland.

#### 4.14.2. Minimal Inhibitory Concentration (MIC)

The MIC of the extracts was determined using a quantitative broth microdilution assay, following Clinical and Laboratory Standards Institute (CLSI) guidelines. Bacterial and fungal cultures grown overnight were diluted 1:10 with fresh brain heart infusion (BHI) broth. A 100 μL volume of the extracts, at an initial concentration of 33 mg/mL, was added to the first well of a 96-well microtiter plate and serially diluted with inoculated broth to achieve final concentrations ranging from 0.25 to 16 mg/mL. Plates were then incubated at 37 °C for 24 h, after which absorbance was measured at 600 nm using an Infinite M200 PRO microplate reader (Tecan, Männedorf, Switzerland). Streptomycin and fluconazole, at concentrations from 0.1 to 250 µg/mL, were used as positive controls for bacteria and fungi, respectively. For streptomycin-resistant bacteria, erythromycin was used. All experiments were conducted in triplicate. The MIC values were defined as the lowest concentration of extract that completely inhibited microbial growth [51].

#### 4.14.3. Minimum Bactericidal/Fungicidal Concentration (MBC/MFC)

The MBC/MFC was determined by plating 100 µL from the last three MIC wells showing inhibited microbial growth onto solid BHI medium. The plates were incubated at 37 °C for 24 h. The MBC/MFC was defined as the lowest concentration resulting in a 99.9% reduction in viable microbial cells. Experiments were run in triplicate.

#### 4.14.4. Inhibition of Biofilm Formation

Biofilm formation was measured following the method described by Merrit et al. [52]. In a 96-well, flat-bottom microplate, 100 µL of BHI containing the extracts (final concentrations of 0.25 to 16 mg/mL) was incubated with the bacteria at 37 °C for 24 h. After incubation, the medium was removed, and wells were washed with water. A 0.1% crystal violet solution was added to each well for 15 min, then removed, and the wells were washed again. The remaining dye was dissolved with 95% ethanol and briefly vortexed. The biomass was quantified by measuring absorbance at 595 nm using an Infinite M200 PRO microplate reader (Tecan, Männedorf, Switzerland). A 10% bleach solution was used as a positive control. Each treatment was carried out in triplicate. The degree of biofilm inhibition was calculated using the following formula [53]:

% biofilm inhibition = (control OD value − treated OD value/control OD value) × 100

### 4.15. Statistical Analysis

The experiments were performed three times, and the data were reported as the average ± standard deviation (SD). All analyses were evaluated in Statistica version 12.0 (StatSoft, Kraków, Poland). The significance of means was examined post hoc by Tukey’s test in conjunction with one-way analysis of variance (ANOVA); *p* < 0.05 was considered significant.

In the analyses using cell lines, statistical analysis of the results was performed using GraphPad Prism 8.4.3 (GraphPad Software, Inc., San Diego, CA, USA). Two-way analysis of variance (ANOVA) and Dunnett’s post hoc intergroup test were used. Statistical significance compared to the control was determined at levels of **** *p* < 0.0001, *** *p* < 0.001, ** *p* < 0.01, and * *p* < 0.05.

For the microbiological tests, because the data were independent and came from more than two groups, statistical analysis was performed using the Kruskal–Wallis test, which does not assume the normality of the data distribution. For statistically significant results, Dunnett’s post hoc intergroup test was carried out. Statistical significance compared to the control was evaluated for levels of *p* < 0.05.

## 5. Conclusions

This research supported the possible application of *Tanacetum parthenium* extracts and disclosed their potency as a natural antioxidant and antimicrobial material. The extract from feverfew flowers was shown to have the highest content of total polyphenols, flavonoids, and phenolic acids, as well as the highest antioxidant potential (DPPH assay, FRAP assay, and in vitro ROS reduction in skin cells). The herb extract, on the other hand, had the highest content of condensed tannins and terpenoids and exhibited the most effective antimicrobial properties against the 12 bacterial and fungal strains tested in the study. Moreover, the hydroethanolic extracts from different parts of *T. parthenium* plants were demonstrated to have potent protective activity for skin cells.

The results of the phytochemical analysis and the in vitro tests of biological properties indicate that feverfew is a safe plant material with notable medicinal potential, which calls for further investigation.

## Figures and Tables

**Table 1:** Characterization of plant material.

Part of Plants	Dried Index	Share of Individual Parts [%]
Aerial parts	3.78 ^b^	100
Leaves	4.53 ^a^	31.7
Flower heads	3.56 ^b^	36.5

Means with different letters in a column differ statistically significantly (*p* < 0.05).

**Table 2:** Content (%) of compounds present in essential oil from *T. parthenium* leaves, herb, and flower heads.

Compound	Leaves	Herb	Flower Heads
α-Pinene	2.34	2.84	0.86
Camphene	7.12	6.57	0.57
p-Cymene	0.89	1.24	0.24
Limonene	1.19	1.10	nd
α-Thujone	0.28	0.22	9.60
Chrysanthenone	0.85	0.56	14.08
Camphor	40.14	38.96	6.30
Borneol	0.65	0.78	2.57
trans-Chrysanthenyl acetate	22.17	25.26	nd
Bornyl acetate	3.04	3.26	0.25
Tetradecane	nd	nd	6.96
Italicene	1.14	0.32	nd
(E)-β-Farnesene	0.04	0.07	1.97
trans-Chrysanthenyl isovalerate	nd	nd	1.43
(Z)-β-Farnesene	nd	0.01	1.07
Viridiflorene	nd	nd	1.47
Bicyclogermacrene	nd	nd	2.31
β-Bisabolene	nd	0.04	3.54
Lauric acid	nd	nd	1.11
Caryophyllene oxide	1.74	1.21	1.11
α-Cadinol	nd	nd	5.84
9-Cedranone	0.90	0.92	1.50
α-Bisabolol	0.63	0.38	2.80
Heptadecane	0.07	0.21	1.14
Pentadecanal	nd	nd	1.78
Methyl myristate	nd	nd	1.63
(Z)-α-Bisabolene epoxide	nd	nd	2.79
4-Methylnonadecane	nd	nd	1.53

nd, not detected.

**Table 3:** Phytochemical composition of extracts from various parts of feverfew.

Extract	Total Polyphenols (mg GAE/mL ± SD)	Total Flavonoids (mg CE/mL ± SD)	Total Phenolic Acids (mg CAE/mL ± SD)	Condensed Tannins (mg DpE/mL ± SD)	Terpenoids (mg LL/mL ± SD)
TLE	53.20 ± 0.17 ^c^	18.10 ± 0.04 ^b^	3.95 ± 0.03 ^c^	16.79 ± 0.07 ^b^	51.33 ± 0.06 ^b^
THE	54.98 ± 0.29 ^b^	17.73 ± 0.10 ^c^	4.51 ± 0.05 ^b^	22.89 ± 0.05 ^a^	54.41 ± 0.06 ^a^
TFE	67.41 ± 0.17 ^a^	19.33 ± 0.11 ^a^	5.10 ± 0.04 ^a^	15.30 ± 0.04 ^c^	41.21 ± 0.09 ^c^

TLE, *T. parthenium* leaf extracts; THE, *T. parthenium* herb extracts; TFE, *T. parthenium* flower head extracts; GAE, gallic acid equivalent; CE, catechin equivalent; CAE, caffeic acid equivalent; DpE, delphinidin; LL, linalool; SD, standard deviation. Means with different letters in columns differ statistically significantly (*p* < 0.05).

**Table 4:** Content of flavonoids and phenolic acids detected using HPLC in extracts from different parts of feverfew.

Compound	Content (mg/mL ± SD)		
	TLE	THE	TFE
Apigenin	0.05 ± 0.00 ^c^	0.15 ± 0.00 ^a^	0.07 ± 0.00 ^b^
Quercetin	0.06 ± 0.00 ^a^	0.05 ± 0.00 ^c^	0.05 ± 0.00 ^b^
Santin	0.32 ± 0.32 ^c^	0.46 ± 0.00 ^a^	0.42 ± 0.00 ^b^
Kaempferol-3-rutinoside	0.40 ± 0.00 ^c^	1.15 ± 0.01 ^a^	0.57 ± 0.00 ^b^
Chlorogenic acid	3.98 ± 0.02 ^c^	4.71 ± 0.01 ^a^	4.13 ± 0.01 ^b^
Neochlorogenic acid	0.31 ± 0.00 ^b^	0.34 ± 0.00 ^a^	0.22 ± 0.00 ^c^
4-O-caffeoyl-quinic acid	0.12 ± 0.00 ^c^	1.59 ± 0.00 ^a^	0.18 ± 0.00 ^b^
3,4-dicaffeoyl-quinic acid	1.28 ± 0.00 ^c^	2.65 ± 0.01 ^a^	1.91 ± 0.01 ^b^
3,5-dicaffeoyl-quinic acid	5.33 ± 0.04 ^c^	8.58 ± 0.01 ^b^	9.89 ± 0.01 ^a^
4,5-dicaffeoyl-quinic acid	0.59 ± 0.00 ^b^	0.72 ± 0.00 ^a^	0.45 ± 0.01 ^c^
Ellagic acid	0.01 ± 0.00 ^b^	0.13 ± 0.00 ^a^	0.00 ± 0.00 ^c^

TLE, *T. parthenium* leaf extracts; THE, *T. parthenium* herb extracts; TFE, *T. parthenium* flower head extracts. Means with different letters in rows differ statistically significantly (*p* < 0.05).

**Table 5:** Antioxidant activity of feverfew extract.

Extract	DPPH (%)	FRAP (mmol/L ± SD)
TLE	64.73 ± 0.44 ^c^	1.34 ± 0.01 ^c^
THE	69.54 ± 0.69 ^b^	1.43 ± 0.03 ^b^
TFE	84.09 ± 0.42 ^a^	1.54 ± 0.02 ^a^

TLE, *T. parthenium* leaf extracts; THE, *T. parthenium* herb extracts; TFE, *T. parthenium* flower head extracts; DPPH, 1,1-diphenyl-2-picrylhydrazyl; FRAP, ferric-reducing antioxidant potential; SD, standard deviation. Means with different letters in columns differ statistically significantly (*p* < 0.05).

**Table 6:** MIC and MBC/MFC values of feverfew extracts and standard drugs in mg/mL.

Strain	TLE		THE		TFE		ST/ER	FL
	MIC	MBC/MFC	MIC	MBC/MFC	MIC	MBC/MFC	MIC	MIC
*S. aureus*	4 ± 0.01	4	4 ± 0.00	4	4 ± 0.02	4	0.001 ± 0.01	-
*S. epidermidis*	4 ± 0.00	8	2 ± 0.03	4	2 ± 0.01	4	0.062 ± 0.04	-
*S. agalactiae*	0.5 ± 0.01	1	1 ± 0.02	2	1 ± 0.05	2	0.001 ± 0.02	-
*S. mutans*	2 ± 0.01	16	2 ± 0.00	4	2 ± 0.00	4	0.004 ± 0.03	-
*S. pyogenes*	4 ± 0.15	4	2 ± 0.02	2	2 ± 0.01	2	0.001 ± 0.02	-
*S. pneumoniae*	0.5 ± 0.01	1	0.25 ± 0.02	0.5	1 ± 0.00	2	0.001 ± 0.02	-
*E. faecalis*	1 ± 0.02	4	2 ± 0.05	4	2 ± 0.010	4	0.004 ± 0.02	-
*E. coli*	0.5 ± 0.03	2	0.5 ± 0.02	2	1 ± 0.03	2	0.015 ± 0.04	-
*P. aeruginosa*	0.5 ± 0.04	2	0.5 ± 0.04	2	2 ± 0.02	4	0.015 ± 0.02	-
*S. sonnei*	1 ± 0.00	2	1 ± 0.01	2	2 ± 0.01	4	0.015 ± 0.02	-
*P. mirabilis*	0.25 ± 0.00	1	0.5 ± 0.02	2	1 ± 0.04	4	0.062 ± 0.07	-
*C. albicans*	0.25 ± 0.00	1	0.25 ± 0.04	1	0.25 ± 0.00	1	-	0.001 ± 0.02

TLE, *T. parthenium* leaf extracts; THE, *T. parthenium* herb extracts; TFE, *T. parthenium* flower head extracts; MIC, minimum inhibitory concentration; MBC, minimum bactericidal concentration; MFC, minimum fungicidal concentration. The positive controls were streptomycin (ST) for bacteria and fluconazole (FL) for fungi. If the bacteria were not susceptible to streptomycin, erythromycin (ER) was used instead; - not used for that strain.

**Table 7:** Percentage inhibition of biofilm formation by feverfew extracts against various microorganisms.

Microorganism	Extract	Extract Concentration (mg/mL)						
		0.25	0.5	1	2	4	8	16
Gram-positive bacteria								
*E. faecalis*	TLE	-	14.4 ± 0.3	49.3 ± 0.1	50.5 ± 0.1	40.6 ± 1.0	-	-
	THE	-	7.9 ± 2.0	-	44.4 ± 0.1	49.3 ± 1.3	45.1 ± 1.6	-
	TFE	-	28.8 ± +1.3	43.8 ± 0.7	44.9 ± 1.0	48.5 ± 0.4	40.4 ± 0.8	26.2 ± 1.0
*S. mutans*	TLE	-	-	-	34.3 ± 10.3	79.8 ± 0.6	73.5 ± 1.1	-
	THE	-	-	-	60.2 ± 9.6	85.8 ± 0.9	83.6 ± 1.8	61.8 ± 2.4
	TFE	-	-	-	-	78.5 ± 1.1	83.8 ± 0.5	77.3 ± 0.9
*S. pneumoniae*	TLE	-	38.4 ± 5.2	64.5 ± 1.0	66.9 ± 0.8	64.7 ± 6.2	36.9 ± 1.2	-
	THE	6.5 ± 8.3	27.4 ± 7.1	58.8 ± 1.2	65.5 ± 1.4	72.9 ± 0.2	68.9 ± 0.4	-
	TFE	-	29.4 ± 6.7	39.9 ± 1.3	67.6 ± 0.6	70.1 ± 0.3	67.8 ± 1.0	56.01 ± 1.4
*S. pyogenes*	TLE	31.7 ± 6.9	58.5 ± 2.1	72.8 ± 0.7	78.3 ± 0.2	75.9 ± 0.5	63.8 ± 0.4	-
	THE	-	-	49.9 ± 6.2	77.9 ± 0.7	76.4 ± 0.2	74.3 ± 1.2	26.9 ± 3.8
	TFE	28.2 ± 8.2	33.8 ± 7.6	40.7 ± 5.9	61.5 ± 1.6	71.5 ± 0.8	75.9 ± 0.0	64.7 ± 0.4
*S. agalactiae*	TLE	-	-	61.4 ± 1.0	66.6 ± 0.6	61.5 ± 0.5	39.9 ± 1.7	-
	THE	-	-	19.7 ± 1.0	66.3 ± 1.3	57.1 ± 3.5	63.3 ± 0.8	-
	TFE	-	-	18.4 ± 6.9	61.1 ± 0.4	64.1 ± 0.4	61.7 ± 0.6	45.5 ± 0.3
*S. aureus*	TLE	51.0 ± 0.5	51.1 ± 0.5	50.7 ± 0.2	48.8 ± 0.5	43.5 ± 0.6	8.5 ± 1.4	-
	THE	52.5 ± 0.2	38.9 ± 2.3	32.6 ± 3.7	49.9 ± 0.4	45.2 ± 0.7	42.6 ± 0.3	-
	TFE	54.6 ± 0.3	54.2 ± 0.3	52.7 ± 0.4	46.9 ± 0.6	54.0 ± 0.5	44.7 ± 0.4	39.4 ± 0.5
*S. epidermidis*	TLE	-	-	-	-	-	-	-
	THE	-	-	-	-	-	-	-
	TFE	-	-	-	-	-	-	-
Gram-negative bacteria								
*S. sonnei*	TLE	32.8 ± 0.3	36.5 ± 0.6	33.5 ± 0.1	24.1 ± 0.4	13.9 ± 0.3	-	-
	THE	33.0 ± 1.0	38.1 ± 0.1	37.4 ± 0.2	32.1 ± 0.0	7.9 ± 5.3	11.2 ± 2.0	-
	TFE	34.6 ± 0.4	35.7 ± 0.2	28.2 ± 1.0	21.7 ± 2.5	35.3 ± 0.9	28.4 ± 0.7	-
*E. coli*	TLE	41.6 ± 0.6	38.3 ± 0.9	47.3 ± 3.1	63.1 ± 0.6	53.8 ± 1.5	40.7 ± 0.4	-
	THE	43.1 ± 0.6	43.5 ± 0.8	39.2 ± 2.2	65.9 ± 0.2	67.3 ± 0.2	61.2 ± 0.1	20.2 ± 0.3
	TFE	26.6 ± 2.3	35.1 ± 1.9	42.3 ± 0.4	54.0 ± 1.2	65.9 ± 0.0	64.0 ± 0.3	53.4 ± 0.6
*P. aeruginosa*	TLE	12.7 ± 6.8	27.1 ± 7.4	74.7 ± 3.1	80.2 ± 0.9	82.4 ± 0.4	77.9 ± 0.9	-
	THE	19.9 ± 2.5	24.6 ± 7.7	80.9 ± 0.9	81.7 ± 1.0	85.0 ± 0.9	84.6 ± 0.2	72.3 ± 1.6
	TFE	16.9 ± 11.3	13.5 ± 8.6	44.0 ± 1.6	70.2 ± 5.3	75.6 ± 2.6	83.2 ± 0.7	79.9 ± 0.7
*P. mirabilis*	TLE	54.8 ± 5.9	68.2 ± 1.6	53.3 ± 2.8	73.1 ± 0.6	73.4 ± 0.5	56.9 ± 1.8	-
	THE	62.8 ± 0.7	69.9 ± 2.2	73.6 ± 1.2	75.4 ± 1.5	75.2 ± 1.0	72.8 ± 0.6	27.5 ± 2.9
	TFE	27.0 ± 4.6	52.7 ± 5.3	71.4 ± 1.6	69.7 ± 0.5	74.6 ± 0.2	76.3 ± 0.8	69.4 ± 0.4
Fungal strain								
*C. albicans*	TLE	72.0 ± 0.7	68.6 ± 0.9	67.4 ± 0.2	67.9 ± 0.1	59.7 ± 0.3	27.4 ± 6.2	-
	THE	74.0 ± 0.0	74.2 ± 0.3	72.4 ± 0.4	69.4 ± 0.8	72.2 ± 0.6	63.3 ± 1.1	15.1 ±1.5
	TFE	59.6 ± 4.3	70.8 ± 0.3	70.4 ± 0.6	71.8 ± 0.3	68.7 ± 0.6	66.2 ± 0.5	56.4 ± 0.4

TLE, *T. parthenium* leaf extracts; THE, *T. parthenium* herb extracts; TFE, *T. parthenium* flower head extracts; -, no inhibition. The positive control was a 10% bleach solution, which resulted in 100% inhibition.

## Data Availability

The data presented in this study are available upon request from the corresponding author.

## Supplementary Materials

The supporting information can be downloaded at: https://www.mdpi.com/article/10.3390/ijms252212179/s1.
