# Supplementary material for: Chemical Composition of Extracts from Various Parts of Feverfew (Tanacetum parthenium L.) and Their Antioxidant, Protective, and Antimicrobial Activities
Source: Int J Mol Sci. 2024 Nov 13;25(22):12179. doi: 10.3390/ijms252212179 (PMC11594288; doi:10.3390/ijms252212179)
Supplement: Supplementary file 1 [file ijms-25-12179-s001.zip › ijms-3257161-supplementary.pdf]

Table S1: The chemical composition of *Tanacetum parthenium* essential oil.

Compound	RT[a]	RI[b]	Content (%)[c]		
			TLE	TFE	THE
α-Tricyclene	8.532	920	0.30 ± 0.01	0.11 ±0.01	0.27 ±0.01
α-Pinene	8.643	932	2.34 ± 0.01	0.86 ±0.02	2.84 ±0.01
Camphene	8.792	948	7.12 ± 0.02	0.57 ±0.02	6.57 ±0.02
Sabinene	9.024	973	0.05 ± 0.01	2.04 ±0.02	0.11 ±0.01
β-Pinene	9.052	976	0.89 ± 0.02	nd	0.75 ±0.01
Myrcene	9.191	991	0.03 ± 0.01	0.16 ±0.01	0.02 ±0.00
α-Terpinene	9.432	1017	0.11 ± 0.01	0.12 ±0.01	0.10 ±0.01
p-Cymene	9.506	1025	0.89 ± 0.02	0.24 ±0.02	1.24 ±0.01
Limonene	9.543	1029	1.19 ± 0.03	nd	1.10 ±0.01
Eucalyptol	9.562	1031	nd	0.84 ±0.01	nd
γ-Terpinene	9.831	1060	0.41 ± 0.02	0.26 ±0.02	0.48 ±0.01
cis-Sabinene hydrate	9.905	1068	0.10 ± 0.01	0.12 ±0.01	0.12 ±0.01
α-Terpinolene	10.100	1089	0.13 ± 0.01	0.06 ±0.01	0.06 ±0.01
Undecane	10.202	1100	0.03 ± 0.00	nd	0.11 ±0.01
Linalool	10.229	1103	0.36 ± 0.01	0.24 ±0.02	0.34 ±0.01
α-Thujone	10.294	1110	0.28 ± 0.01	9.60 ±0.02	0.22 ±0.01
β-Thujone	10.359	1117	0.60 ± 0.01	0.65 ±0.02	0.89 ±0.01
Chrysanthenone	10.443	1126	0.85 ± 0.01	14.08 ±0.03	0.56 ±0.01
3-Thujol	10.554	1138	nd	0.22 ±0.01	nd
trans-Pinocarveol	10.610	1144	0.45 ± 0.01	0.41 ±0.02	0.40 ±0.01
Camphor	10.684	1152	40.14 ± 0.02	6.30 ±0.03	38.96 ±0.03
cis-Chrysanthenol	10.804	1165	0.85 ± 0.01	0.09 ±0.01	0.86 ±0.01
Borneol	10.841	1169	0.65 ± 0.01	2.57 ±0.02	0.78 ±0.01
Terpinen-4-ol	10.934	1179	0.87 ± 0.02	0.71 ±0.01	0.95 ±0.01
α-Terpineol	11.073	1194	0.33 ± 0.02	0.11 ±0.01	0.18 ±0.01
1-Dodecene	11.092	1196	nd	nd	0.10 ±0.01
Myrtenol	11.120	1199	0.61 ± 0.01	nd	0.41 ±0.01
Decanal	11.175	1205	0.26 ± 0.01	nd	0.22 ±0.01

cis-Verbenone	11.250	1213	0.54	± 0.01	nd		0.36	±0.01
(E)-Carveol	11.352	1224	0.17	± 0.01	nd		0.21	±0.01
trans-Chrysanthenyl acetate	11.491	1239	22.17	± 0.04	nd		25.26	±0.04
Hexyl isovalerate	11.565	1247	0.59	± 0.02	nd		0.62	±0.01
cis-Chrysanthenyl acetate	11.732	1265	0.42	± 0.02	nd		0.24	±0.01
Bornyl acetate	11.945	1288	3.04	± 0.04	0.25	±0.02	3.26	±0.02
trans-Pinocarvyl acetate	12.001	1294	nd		0.09	±0.01	nd	
cis-Pinocarvyl acetate	12.196	1315	0.25	± 0.01	nd		0.47	±0.01
neo-Verbanol acetate	12.316	1328	0.17	± 0.01	nd		0.25	±0.01
γ-Pyronene	12.427	1340	nd		0.01	±0.01	nd	
α-Cubebene	12.520	1350	0.34	± 0.01	0.10	±0.01	0.15	±0.01
Eugenol	12.604	1359	0.48	± 0.01	nd		0.29	±0.01
Nerol acetate	12.678	1367	0.06	± 0.01	nd		0.05	±0.01
Capric acid	12.743	1374	0.35	± 0.01	nd		0.32	±0.01
β-Bourbonene	12.882	1389	nd		0.70	±0.01	0.04	±0.01
β-Elemene	12.937	1395	0.41	± 0.01	nd		0.05	±0.01
Tetradecane	12.975	1399	nd		6.96	±0.02	nd	
Italicene	13.012	1403	1.14	± 0.01	nd		0.32	±0.01
α-Cedrene	13.132	1416	nd		0.34	±0.02	nd	
β-Caryophyllene	13.225	1426	0.50	± 0.02	0.92	±0.02	0.35	±0.01
β-Copaene	13.271	1431	nd		0.14	±0.01	nd	
β-Cubebene	13.308	1435	nd		0.09	±0.01	nd	
(Z)-β-Santalene	13.447	1450	nd		0.33	±0.01	nd	
(E)-β-Farnesene	13.559	1462	0.04	± 0.01	1.97	±0.02	0.07	±0.00
Undecanoic acid	13.605	1467	nd		0.43	±0.01	0.08	±0.00
γ-Gurjunene	13.642	1471	nd		0.88	±0.01	nd	
β-Chamigrene	13.689	1476	nd		0.15	±0.01	nd	
trans-Chrysanthenyl isovalerate	13.735	1481	nd		1.43	±0.01	nd	
Germacrene D	13.791	1487	nd		0.30	±0.01	nd	
(Z)-β-Farnesene	13.828	1491	nd		1.07	±0.02	0.01	±0.00
Viridiflorene	13.846	1493	nd		1.47	±0.01	nd	
Pentadecane	13.911	1500	0.04	± 0.01	nd		0.09	±0.01

Bicyclogermacrene	13.930	1502	nd	2.31 ±0.02	nd
β-Bisabolene	14.050	1515	nd	3.54 ±0.01	0.04 ±0.00
β-Sesquiphellandrene	14.106	1521	0.05 ± 0.01	0.14 ±0.00	0.11 ±0.00
δ-Cadinene	14.171	1528	nd	0.74 ±0.01	nd
trans-Calamenene	14.208	1532	nd	0.11 ±0.01	nd
Cadina-1,4-diene	14.273	1539	nd	0.36 ±0.01	nd
Germacrene B	14.431	1556	nd	0.14 ±0.01	nd
β-Calacorene	14.477	1561	0.05 ± 0.01	0.16 ±0.01	0.02 ±0.01
(E)-Nerolidol	14.533	1567	0.18 ± 0.01	1.48 ±0.02	0.36 ±0.01
Lauric acid	14.616	1576	nd	1.11 ±0.01	nd
Chrysanthenyl hexanoate	14.681	1583	nd	0.57 ±0.01	nd
Spathulenol	14.710	1586	0.36 ± 0.01	0.50 ±0.01	0.26 ±0.02
Caryophyllene oxide	14.765	1592	1.74 ± 0.01	1.11 ±0.01	1.21 ±0.02
Hexadecane	14.867	1603	0.40 ± 0.02	0.56 ±0.01	0.20 ±0.01
Humulene epoxide II	14.959	1613	nd	0.20 ±0.01	0.09 ±0.01
1,10-di-epi-Cubenol	15.015	1619	0.35 ± 0.01	0.53 ±0.02	0.20 ±0.01
Isospathulenol	15.163	1635	nd	0.72 ±0.01	nd
β-Eudesmol	15.275	1647	0.93 ± 0.01	0.44 ±0.01	0.94 ±0.02
τ-Cadinol	15.339	1654	nd	0.89 ±0.02	nd
α-Cadinol	15.460	1667	nd	5.84 ±0.02	nd
9-Cedranone	15.469	1668	0.90 ± 0.01	1.50 ±0.01	0.92 ±0.01
α-Bisabolol	15.590	1681	0.63 ± 0.01	2.80 ±0.03	0.38 ±0.01
epi-α-Bisabolol	15.664	1689	nd	0.89 ±0.02	nd
β-Sinensal	15.711	1694	nd	0.70 ±0.01	nd
1- -Heptadecene	15.738	1697	0.25 ± 0.01	nd	0.01 ±0.00
Heptadecane	15.785	1702	0.07 ± 0.01	1.14 ±0.02	0.21 ±0.01
Pentadecanal	15.924	1717	nd	1.78 ±0.01	nd
(E,E)-Farnesol	15.989	1724	nd	0.92 ±0.01	nd
Methyl myristate	16.026	1728	nd	1.63 ±0.01	nd
(Z)-α-Bisabolene epoxide	16.063	1732	nd	2.79 ±0.02	nd
Bisabolol oxide A	16.267	1754	nd	0.47 ±0.02	nd
Myristic acid	16.397	1768	0.32 ± 0.01	0.18 ±0.01	0.51 ±0.02

(E)-α-Atlantone	16.499	1779	0.25 ± 0.01	0.19 ±0.01	0.26 ±0.02
1-Octadecene	16.619	1792	nd	0.09 ±0.01	nd
7-(2,4-Hexadiene Ylidene))-1,6- -dioxaspiro[4.4]nona-2,8-diene	16.823	1814	0.08 ± 0.01	nd	0.08 ±0.01
1-Hexadecanol	17.454	1882	0.13 ± 0.01	nd	0.13 ±0.01
Nonacosane	17.640	1902	0.77 ± 0.01	nd	0.43 ±0.01
Methyl palmitate	17.871	1927	nd	0.16 ±0.01	nd
m-Camphorene	18.150	1957	nd	0.54 ±0.01	nd
4-Methylnonadecane	18.187	1961	nd	1.53 ±0.01	nd
Palmitic acid	18.298	1973	0.23 ± 0.01	nd	0.21 ±0.02
Ethyl palmitate	18.391	1983	nd	nd	0.10 ±0.01
Eicosane	18.604	2006	nd	0.05 ±0.01	nd
1-Heneicosene	19.429	2095	nd	0.25 ±0.01	nd
Ethyl linoleate	19.995	2156	nd	0.35 ±0.01	nd
1-Tricosene	21.284	2295	nd	0.60 ±0.01	nd
1-Pentacosene	23.13	2494	nd	0.79 ±0.02	nd
Pentacosane	23.213	2503	0.18 ± 0.01	nd	0.10 ±0.00
1-Heptacosene	25.022	2698	nd	0.20 ±0.01	nd
Total identified (%)			98.37 ± 0.04	99.37 ±0.35	97.53 ±0.67
Monoterpene hydrocarbons			13.83 ±0.02	5.78 ±0.07	13.99 ±0.06
Oxygenated monoterpenes			74.55 ±0.02	43.61 ±0.06	76.57 ±0.09
Sesquiterpene hydrocarbons			3.28 ±0.02	17.75 ±0.03	1.64 ±0.10
Oxygenated sesquiterpenes			5.33 ±0.02	27.40 ±0.04	4.62 ±0.02
Diterpenoids			nd	2.47 ±0.10	nd
Others compounds			1.39 ±0.01	2.42 ±0.01	1.05 ±0.03

[a] RT, retention time (minute); [b] RI, the Kovats retention index; [c] percentage composition of a compound; nd, not detected; TLE, *T. parthenium* leaves extracts; THE, *T. parthenium* herb extracts; TFE, *T. parthenium* flower heads extracts
